# Photosynthesis in Hydrogen-Dominated Atmospheres

**DOI:** 10.3390/life4040716

**Published:** 2014-11-18

**Authors:** William Bains, Sara Seager, Andras Zsom

**Affiliations:** 1 Department of Earth, Atmospheric and Planetary Sciences, Massachusetts Institute of Technology, 77 Massachusetts Ave., Cambridge, MA 02139, USA; E-Mails: seager@mit.edu (S.S.); zsom@mit.edu (A.Z.); 2 Department of Physics, Massachusetts Institute of Technology, 77 Massachusetts Ave., Cambridge, MA 02139, USA; 3 Rufus Scientific Ltd, 37 The Moor, Melbourn, Royston, Herts SG8 6ED, UK

**Keywords:** photosynthesis, exoplanet, biomass, hydrogen atmosphere

## Abstract

The diversity of extrasolar planets discovered in the last decade shows that we should not be constrained to look for life in environments similar to early or present-day Earth. Super-Earth exoplanets are being discovered with increasing frequency, and some will be able to retain a stable, hydrogen-dominated atmosphere. We explore the possibilities for photosynthesis on a rocky planet with a thin H_2_-dominated atmosphere. If a rocky, H_2_-dominated planet harbors life, then that life is likely to convert atmospheric carbon into methane. Outgassing may also build an atmosphere in which methane is the principal carbon species. We describe the possible chemical routes for photosynthesis starting from methane and show that less energy and lower energy photons could drive CH_4_-based photosynthesis as compared with CO_2_-based photosynthesis. We find that a by-product biosignature gas is likely to be H_2_, which is not distinct from the hydrogen already present in the environment. Ammonia is a potential biosignature gas of hydrogenic photosynthesis that is unlikely to be generated abiologically. We suggest that the evolution of methane-based photosynthesis is at least as likely as the evolution of anoxygenic photosynthesis on Earth and may support the evolution of complex life.

## 1. Introduction

Photosynthetic organisms dominate Earth’s biosphere. Light is by far the most abundant source of chemical energy on the surface of the Earth, so any form of life that evolves the ability to capture light energy will be able to out-compete its non-photosynthetic sister species, at least for growth on the surface of the planet. Light will be an abundant and accessible energy source on the surface of any planet with a sufficiently thin atmosphere. Understanding the chemistry of photosynthesis is therefore important to understanding the possible biospheres on other worlds and specifically to predicting life’s possible atmospheric signatures on those worlds.

In this paper, we investigate, for the first time to our knowledge, how life can use light energy to capture atmospheric carbon in an environment that is dominated by hydrogen and methane. We specifically address the energy requirements, possible photon wavelength requirements and whether such photosynthesis would generate as distinct a biosignature as oxygenic photosynthesis makes on Earth.

### 1.1. Role and Evolution of Photosynthesis

The ability to capture the energy of electromagnetic radiation has evolved at least three times on Earth, with mechanisms mediated by chlorophyll [1,2,3], rhodopsin-like proteins [4,5], which can capture light energy for biosynthesis [6,7], and fungal melanin-based systems that capture short wavelength light and ionizing radiation and use at least some of the captured energy to power ATP synthesis [8,9,10]. Several lines of fossil, trace chemical and genetic evidence suggest that chlorophyll-based photosynthesis had evolved by within 500 My of the end of the Late Heavy Bombardment period, when Earth’s surface became continuously habitable [1,11]. The carbon isotope ratio at 3.5 Gya is interpreted by some as evidence that microbial RuBisCO-based carbon fixation occurred at this early date [12], although this is not evidence that oxygenesis evolved at that early date, as the evolution of RuBisCO and the evolution of oxygenesis could have occurred at very different times. The speed with which photosynthesis evolved on Earth suggests that analogous biochemistry might be expected to evolve on other inhabited worlds.

Photosynthesis is a combination of two processes—the capture of light energy and the use of that energy to drive thermodynamically unfavorable redox reactions. The redox reactions are needed to build biomass [13]. Living organisms build biomass by capturing environmental carbon, nitrogen, oxygen, phosphorus and sulfur and incorporating those elements into the complex molecules of biochemistry. We note that a minority of organisms, including ourselves, build biomass by eating other organisms (heterotrophy: “eating others”). However, clearly, the heterotrophic lifestyle cannot dominate the biosphere.

Photosynthesis that captures light energy and CO_2_ to build biomass, generating free oxygen as a waste product, dominates Earth’s modern biosphere [14,15]. In textbooks, terrestrial photosynthesis is usually illustrated by the chemistry needed to build carbohydrates (compounds of the general formula CH_2_O) from carbon dioxide (CO_2_). This requires energy input. In terrestrial plants, the energy captured from photons is used to power the reaction:

CO_2_ + H_2_O + h*v* →CH_2_O + 2[O] (1)

where [O] represents an oxidized by-product and h*v* represents the energy of photons of frequency *v*. Oxygenic photosynthesis produces molecular oxygen as a by-product, so the reaction becomes:

CO_2_ + H_2_O + h*v*→CH_2_O + O_2_ (2)

We note that here, and elsewhere, we write these equations as if they were simple chemical reactions, in this case, one CO_2_ molecule and one H_2_O molecule reacting to form formaldehyde and oxygen. This is of course a gross over-simplification of the complex molecular machinery of photosynthesis. However it does illustrate the overall stoichiometry (input and output) of the process, from which the minimum energy input to make the process happen can be calculated.

### 1.2. Photosynthesis beyond Earth

No life, including photosynthesizing life, has been found beyond Earth. Theoretical studies of exoplanet photosynthesis have primarily addressed whether planets orbiting around different stars can have surface illumination consistent with terrestrial photosynthesis. Different groups have considered, at various levels of detail: planets illuminated by Sun-like stars at differing evolution stages and for Earth’s atmosphere’s assumed evolution over time [16,17]; F, K stars [18]; M stars [19,20,21]; multiple star systems [22]; tidally locked and 3:2 spin-orbit resonance planets [23]; and moons of giant planets [24]; see also the general reviews in [21,25]. For photosynthetic signals, all have assumed basically terrestrial biochemistry. Kiang *et al.* have gone into detail about the likely pigment absorption needed for life living under stars with a radiation output different from the Sun, but assume throughout that photosynthesis is used to drive CO_2_ reduction [26]. Many studies relate the stars’ spectrum to the need to balance photosynthetically-active radiation (green and longer) with damaging radiation (blue and shorter) [20,21].

The impact of photosynthesis on the atmosphere and the oxidation of the Earth has been the subject of extensive study, summarized in [27]. Other studies examine the impact of the evolution and the consequences of oxygenic photosynthesis on Earth [1,16,17,25,28,29,30,31]. These studies are all of terrestrial photosynthesis, *i.e.*, photosynthesis that oxidizes the environment in order to build biomass. The role of photosynthesis in allowing Earth to develop an abundant biosphere [25,30,32] and its potential role in enabling complex life based on aerobic metabolism [2,30] have also been studied.

Biosignatures generated from a photosynthetic biosphere like Earth’s, primarily oxygen and ozone, but also the oxygen/methane mix, have been discussed extensively (e.g., [33,34,35,36,37,38]). These obviously all assume O_2_ production. The “red edge” signature [37,39,40] may [36], or may not [41], be related to the chemistry of oxygenic photosynthesis. (In any case, the red edge is a less reliable signature than O_2_ and O_3_ [42,43].)

The previous studies cited above do not address the photosynthetic chemistry that might happen on a planet with a highly reducing atmosphere, the subject of this paper.

### 1.3. Hydrogen-Rich Rocky Exoplanets

All of the studies above assume that a habitable planet has an oxidized atmosphere that contains CO_2_, N_2_, H_2_O and only trace levels of CH_4_ or H_2_. However a reduced atmosphere planet, with significant H_2_ content, is possible.

Some Super-Earth planets (loosely defined as rocky planets of up to 10 Earth masses) may be able to retain hydrogen in their atmospheres. Planets can capture hydrogen from the stellar nebula during formation [44] or outgas hydrogen after formation. Planets built from materials containing water-rich materials are expected to release H on early degassing [45,46]. Planets that retain a primordial H_2_-rich envelope during the accretion phase [44] and that have high enough surface gravity, low enough temperature and/or high enough magnetic fields should be able to retain the hydrogen in their atmosphere (e.g., [47]). The planet must have a stable surface that is compatible with the existence of a surface liquid to be habitable; the most plausible liquid to support life is water.

Planets with a very dense gaseous envelope (“sub-Neptunes”) will have a surface too hot for liquid water, if they have a defined surface at all (Seager and Rogers in preparation). However rocky planets with a thin, hydrogen-dominated atmosphere can have a surface temperature compatible with liquid water. H_2_:H_2_ collision-induced absorption (CIA) of near-infrared (NIR) light provides a strong greenhouse effect [47,48], which can mean that such planets have surface temperatures compatible with liquid water well outside the conventional “habitable zone”. Thus, the habitable zone for super-Earths with a H_2_-dominated atmosphere can be much more extensive than that for truly Earth-like planets (reviewed in [49]). However, there are limits to the extension of the habitable zone for a planet with an H_2_-dominated atmosphere. In general, the atmospheric greenhouse effect caused by H_2_-H_2_ CIA will increase with increasing atmospheric depth, but the attenuation of light reaching the surface will also increase with atmospheric depth. For a very dense atmosphere, surface photosynthesis will not be possible, because the surface will be dark. We return to this in Section 3.6 below.

The chemistry of the surface environment on an H_2_-dominated planet may be very different from that on an oxidized planet, such as the early Earth. A different environment will require a different chemistry to capture environmental carbon into biomass. This paper addresses the question of what that chemistry might be and what combination of atmospheric and orbital environment might support the life that executes such chemistry.

## 2. Approach, Methods and Data Sources

### 2.1. Overall Approach

We approach the problem of the nature of photosynthesis on a hydrogen-dominated world as follows. First, we identify what the dominant environmental source of carbon that is to be captured by photosynthesis will be on such a world, and from this, we identify the fundamental transitions that life must perform to change this dominant carbon species into biomass. In a second step, we identify the environmental chemicals that could participate in the chemical transitions necessary for photosynthesis. The third stage of the analysis is to identify how much energy is needed to drive the chemical transformations that we have identified for comparison with terrestrial photosynthesis. The fourth stage is to estimate the energy of the photons necessary to provide that energy, given the nature of the specific chemicals involved. Lastly we map these requirements of chemical input, energy input and photon wavelength onto possible planetary environments. We now describe the methods for each of these steps in more detail.

The composition of the atmosphere of the exoplanet is taken as a starting point for our study. We assume a hydrogen-dominated atmosphere, as described in [50].

We identify the sources of carbon in each environment from the photochemistry and geochemistry of that environment. Photochemical models of a wide range of planetary atmospheres have been well studied in the past [50,51,52], and we take these results as our starting point. The chemistry of life has to be of intermediate redox state [13], and so simple arguments show how life must take oxidized or reduced carbon and convert it to biomass of any sort.

We identify environmental chemicals by assuming that the crust of an exoplanet has a similar elementary composition to that of the Earth. Thus, we assume that elements, like silicon, which are common on the Earth, will be common on a rocky exoplanet, and elements, like gold, which are rare on Earth, will be rare on a rocky exoplanet.

The calculation of the energy needed to build biomass is complicated, and so we discuss this in a separate section below. Photon energies are calculated assuming that a single photon is absorbed to elevate a single electron to drive a one electron reaction, and we follow the method used to estimate the wavelength of light necessary to drive terrestrial photosynthesis in [53]. Further details of these calculations are given in Section 3.5.

Lastly, we integrate the total energy demands and the photon energy requirements into a planetary model by modelling the atmospheric radiation flux to the surface through atmospheres of different density, on a planet with orbital parameters such that its average surface temperature is clement.

### 2.2. Energy Calculations

We address the problem of calculating the energy needed to make a set of chemicals selected from an effectively infinite chemical space in three stages. The first stage generates a systematic description of all of the molecules in a chemical space of a particular size (the size has to be limited, as there is an infinite number of molecules in the chemical space if the size is not limited). The second stage estimates the standard free energy of formation (ΔG^0^) of the chemicals in the chemical space. The standard free energy is the energy needed to make the molecules from their elements and is characteristic of the molecule and does not depend on how you make it. The third stage then calculates the energy actually needed to turn a set of environmental molecules into each of the molecules in the chemical space. This is the energy actually needed to build biomass in a real environment.

The chemical space of possible biochemicals is calculated as follows. We used a SMILES-based combinatorial approach to building molecular structures, implemented in the program COMBIMOL [13], to generate a list of nearly [54] all plausible carbon-based chemicals of a defined size. For this work, we chose molecules of up to 9 non-hydrogen atoms, made of C, N, O, S in oxidation states −2, 0, 2 or 4, and P in oxidation state +5. Rings of 4 or more atoms were allowed. This resulted in a set of 1,987,593 structures.

Many of the structures generated by COMBIMOL represent chemicals whose free energy of formation (ΔG^0^) is not known. Some of these chemicals are highly-strained molecules, such as fused cyclobutane rings, azetes, *etc*. While plausible potential molecules, their free energies of formation have not been measured. The free energy of formation of the molecular structures was therefore calculated using semi-empirical quantum mechanical methods. Because these methods are very computationally intensive (requiring several minutes of PC processor time per molecule), we calculated the ΔG^0^ for a sample subset of molecules and then extrapolated this to the entire set of 1,987,593 structures.

The ultimate goal of this calculation is to compare the energies of synthesis of molecules in different redox environments (*i.e.*, environments with or without a large amount of hydrogen). We therefore generated the sample subset of chemicals on which to perform ΔG^0^ calculations as follows. We define the state of oxidation or reduction of a molecule in structural terms, using the redox ratio (*Rr*), as described in [13]. In brief, *Rr* is a simple measure of the degree of saturation of a molecule with hydrogen. *Rr* is defined as:

(A) $$Rr=1-\frac{\sum^{\text{​}}S_{a}}{\sum^{\text{​}}S_{h}}$$

where S_a_ is the actual number of hydrogen atoms bonded to an atom and S_h_ is the number that could be bonded if the atom were fully reduced with hydrogen. This is a simple measure of reduction that is suited to comparing molecules’ redox state in a hydrogen-dominated environment. We note that this does not relate directly to standard electrode potentials or other, energetic measures of redox state. It is simply a convenient classification based on chemical structure alone.

A sample set of 2275 molecules were randomly selected from the chemical space to provide up to 30 examples of all of the *Rr* values present in the whole molecule set. Thus, for example, only one molecule with an *Rr* = 0.333 was present in the complete chemical space—propane—and so this was included in the calculation subset, but many molecules of the formula C_6_H_15_N, with an *Rr* = 0.444, are present, so a random subset of 30 of these were included. For each of the molecules in this subset, the enthalpy of formation was calculated using MOPAC [55] version 2009 running in CambridgeSoft ChemOffice 12.0 under a site license to MIT, using the “Minimize energy” function with RMS = 0.0095, the open shell wavefunction and the PM3 and PM6 methods. PM3 and PM6 gave very similar results for almost all molecules (r^2^ = 0.955): for this study, the average of the PM3 and PM6 estimates of ΔH were used, and ΔS was estimated from molecular formulae following [56].

The distribution of standard free energy of the formation of all of the components of the chemical space were estimated from the sample of 2275 molecules as follows. Molecules were binned into 20 *Rr* bins, and the distribution of ΔG values for each of those bins was calculated for the 2275-molecule sample. The number of molecules in the complete set of 1,987,593 molecules that were in each *Rr* bin was counted. The same energy distribution was assumed to apply to all of the molecules in an *Rr* bin as applied to the smaller sample.

## 3. Results

We present the results of our analysis of the possibilities for photosynthesis in an atmosphere dominated by hydrogen.

### 3.1. Carbon-Containing Species in an H_2_-Dominated Atmosphere

We start with a summary of our initial assumptions about the redox state of the principle elements of life in an oxidizing and a reducing environment. We start with the discussion of atmospheric carbon.

Carbon can be present as CO_2_, CO or CH_4_ in a planet’s atmosphere, as well as other, minor species. In an oxidized atmosphere under stellar UV, CH_4_ has a short half-life and is rapidly converted to CO or CO_2_ by UV photolysis followed by a reaction with other atmospheric species. By contrast, in an atmosphere with >70% hydrogen, methane is extremely long lived. This is because photolysis of methane produces CH_3_· radicals, which in an H_2_-dominated atmosphere, predominantly react with hydrogen atoms or molecules to regenerate CH_4_ [52,57], and because there is no surface chemistry that absorbs CH_4_ into the surface in wet or dry deposition processes at temperatures below 100°C [50]. In a reducing atmosphere, photochemical reaction of CO_2_ is inefficient [50]; photochemical reaction of CO with hydrogen is also slow [58], and both reaction pathways end in CH_4_. CO_2_ is also removed from the atmosphere by conversion to carbonate, at a rate that depends on surface chemistry.

Thus, while small amounts of CO_2_ and CO might be converted to CH_4_ and some CO_2_ may be removed from the atmosphere by wet deposition to the surface, the ratio of carbon species in the atmosphere is likely to be dominated by their outgassing ratio. The ratio of CH_4_ to CO_2_ outgassed will depend on mantle and surface chemistry. Mantle chemistry is unlikely to generate substantial CH_4_ fluxes unless the mantle is extremely reduced. Surface hydrothermal chemistry on Earth can convert up to 10% of emitted carbon into CH_4_ (e.g., [59,60]), but more often, hydrothermal gases contain less than 1% of their carbon as CH_4_. Thus, a rocky planet with an atmosphere containing >70% H_2_ could have substantial CH_4_ content, but the ratio of CO_2_ to CH_4_ will depend on the specifics of the planet.

However, in the presence of life, we expect the atmosphere to contain far more CH_4_, for the following reason. The reaction:

CO_2(aq)_ + 4 H_2(aq)_→CH_4(aq)_ + 2 H_2_O_(aq)_ (3)

yields 193 kJ/mol standard free energy at 25 °C. This is the reaction that terrestrial methanogens use to capture energy. The reaction would yield 10 kJ/mol (the minimum free energy usable by terrestrial methanogens [61,62]) in water under an atmosphere containing 10^−72^ as much CO_2_ as CH_4_ and 70% H_2_ at 1 bar. Thus, life could use the energy released by the reduction of CO_2_ to CH_4_ until essentially all of the CO_2_ was consumed. Reaction of CO_2_ and H_2_ would represent a ubiquitous source of energy for life on a world with an H_2_-dominated atmosphere. Even in the relatively oxidized atmosphere of the early Earth, it is plausibly argued that the atmosphere might have contained roughly equal amounts of methane and carbon dioxide [63].

We can therefore consider three scenarios for the atmosphere of a rocky planet with an H_2_-rich (>70%) atmosphere.

(1) Minimal methane: atmospheric carbon present almost entirely as CO or CO_2_, because there is no life to generate methane, and only a small amount of methane is outgassed.
(2) Methane and carbon dioxide: atmospheric carbon present as methane and carbon dioxide in a ratio of between 1:10 and 10:1, because of higher rates of outgassing of methane than on Earth and/or some limited life.
(3) High methane: atmospheric carbon present almost entirely as methane, because carbon is outgassed almost entirely as methane (an unlikely scenario, but possible) and/or because life is abundant.

Because this paper is concerned with possible photosynthesis reactions on an inhabited planet, we will consider the third of these scenarios, one in which atmospheric carbon is present primarily or exclusively as methane. We note that the third scenario is itself indicative of the presence of life, *i.e.*, the presence of methane and the absence of carbon dioxide is a weak biosignature in its own right, even in an H_2_-dominated atmosphere.

The redox states of the other main elements used by terrestrial life to build biomass are listed in Table 1. Only sulfur has a different redox state from Earth. Sulfur is readily reduced by environmental reducing agents, and so would be expected to be present as sulfides on a planet whose surface and atmosphere was dominated by hydrogen [51].

**Table 1:** Environmental redox states of biochemical elements.

Element	Dominant Environmental Form	
	Oxidized Environment	Reduced Environment
C	CO_2_	CH_4_
S	SO_4_^2−^	H_2_S
N	N_2_	N_2_
P	PO_4_^2−^	PO_4_^2−^
O	H_2_O	H_2_O

Assumed chemical form of the major elements used in biochemicals in oxidizing and reduced environments.

### 3.2. Overall Reaction for Photosynthesis in a CH_4_/H_2_ Atmosphere

Life needs to build biomass (more life) from environmental chemicals. Environmental chemicals will not be in an oxidation state that is suitable for direct incorporation into biochemicals [13]. Therefore, life has to carry out redox chemistry to convert environmental chemicals into biochemicals. As reviewed in Section 1.1, terrestrial life builds biomass from CO_2_. In this paper, we are concerned with how life builds biomass from CH_4_ in a reducing environment.

Methane is the most reduced form of carbon, so in order to build complex molecules, it must be oxidized. In a reducing environment, oxidizing methane will require energy, which here we assume comes from light. The analogous reaction to Reaction (1) above is therefore

CH_4_ + H_2_O + h*v*→CH_2_O + 4[H] (4)

A simple version of this reaction would be one that generated hydrogen gas, thus:

CH_4_ + H_2_O + h*v*→CH_2_O + 2H_2_ (5)

By analogy with the term “oxygenic” photosynthesis, we refer to Reaction (5) as “hydrogenic photosynthesis”.

Reaction (5) above is written as a process whose net output is hydrogen, just as photosynthesis based on CO_2_ is written in Reaction (2) as producing oxygen. On Earth, the dominant photosynthetic reaction does produce oxygen gas as a by-product. However, a range of terrestrial photosynthetic reactions are known that do not produce oxygen, but rather use other electron donors to reduce CO_2_, and these are termed anoxygenic photosynthesis.

Anoxygenic photosynthesis on Earth produces oxidized products other than molecular oxygen, for example oxidized iron (in the form of hydroxides under neutral pH conditions [64]):

CO_2_ + 4 Fe(OH)_2_ + 3H_2_O + h*v* → CH_2_O + 4Fe(OH)_3_ (6)

Photosynthetic bacteria also oxidize sulfide to sulfate and hydrogen to water (reviewed in [38]). Both the oxidation of sulfur to sulfate and the oxidation of hydrogen are energy-releasing reactions on Earth, and so the light capturing apparatus is being used in these organisms as a supplement to chemosynthetic processes and not as the primary energy source.

We might therefore suspect that life on a hydrogen-dominated world might evolve photosynthetic chemistry that does not produce hydrogen (“anhydrogenic” photosynthesis) if suitable reactions are available.

Anhydrogenic photosynthesis might dominate if: (1) the mechanisms for hydrogen evolution had not evolved; (2) light energy was a limiting resource, and reactions that required less energy were available; (3) a source of hydrogen atoms to build into H_2_ was limiting; or (4) some combination of these. The next sections address the overall energetics of hydrogenic photosynthesis and the relative energetics of hydrogenic *vs.* anhydrogenic photosynthesis.

### 3.3. Energy Requirements for Biomass Building in a Reduced Environment

We now address the overall energy needed to build biomass from methane in a hydrogen-dominated environment, to demonstrate the plausibility of hydrogenic photosynthesis and to provide a comparison with the productivity of terrestrial photosynthesis.

**Figure 1:**
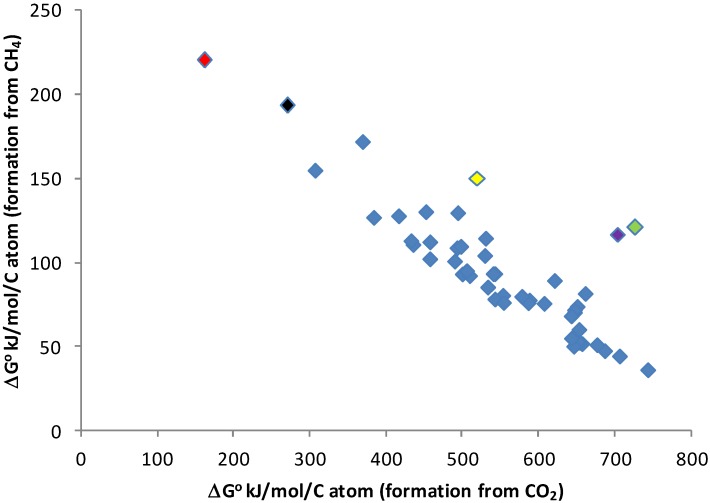
Energy of the synthesis of sample compounds. Comparison of Gibbs free energy of the synthesis of 49 terrestrial metabolites from CO_2_, H_2_O, SO_4_^2−^ and N_2_ (X-axis) or CH_4_, H_2_O, H_2_S and N_2_ (Y-axis). Free energy is for unionized compounds in aqueous solution, at 25 °C, except for octane, nonane, decane, undecane and hexadecane, which are calculated as liquids, because of their very low solubility in water. Data from [65]. Metabolites (with coloring to identify outliers) are formic acid (black point), acetic acid, glycolic acid, propanoic acid, lactic acid, butanoic acid, pentanoic acid, benzoic acid, oxalic acid (red point), malonic acid, succinic acid, glutaric acid, methanol (purple point), ethanol, propanol, 2-propanol, butanol, pentanol, ethane, propane, butane, pentane, octane, nonane, decane, undecane, hexadecane, toluene, ethylbenzene, alanine, arginine, asparagine, aspartic acid, cysteine (green point), glutamic acid, glutamine (yellow point), glycine, histidine, isoleucine, leucine, lysine, methionine, phenylalanine, proline, serine, threonine, tryptophan, tyrosine and valine.

Comparison of Gibbs energies of formation of CO_2_ (gas ~ −394 kJ/mol, aq ~−385 kJ/mol) and CH_4_ (gas ~ −50 kJ/mol, aq ~ −35 kJ/mol) [65] shows that any reaction involving CO_2_ as the C-bearing reactant will almost always have a more positive Gibbs energy of reaction than a similar reaction with CH_4_ as the reactant. The quantitative difference between the reactions will depend on the products of the reaction, as illustrated in Figure 1. On average, for the set of chemicals in Figure 1, making the chemical from CH_4_ takes ~20% the energy needed to make it from CO_2_. This suggests that building biomass in a CH_4_/H_2_-dominated environment would require only ~20% of the energy needed in our CO_2_-dominated environment.

The calculation we have made above of the energy needed to make a set of molecules from CO_2_ and from CH_4_ does not provide a strong argument for the comparative energetics of building biomass in an oxidized and a reduced environment, respectively. The calculation assumes that the biomass made by hydrogenic photosynthesis has the same chemical composition as life made by oxygenic photosynthesis. This is not necessarily true. The components of terrestrial metabolism are more oxidized than a random selection of molecules from the chemical space would be expected to be [13,66], which might be because life evolved to minimize the energy needed to convert CO_2_ into biomass. If this were so or if evolution took another path on an exoplanet for other reasons, then the biomass being built by hydrogenic photosynthesis could be different from that built on Earth by oxygenic photosynthesis.

We confirm that biomass synthesis in a CH_4_/H_2_-dominated environment would require less energy than in a CO_2_-dominated environment as follows. We argue that life has to build a diverse subset of chemicals from the chemical space of all organic chemicals. We assume that the fraction of chemical space that life has to explore to build that diverse set is the same in oxidizing or reducing environments. We therefore calculate how much energy is needed to build two different sets of molecules that represent a subset of all molecules in the chemical space from which biochemistry is selected.

**Figure 2:**
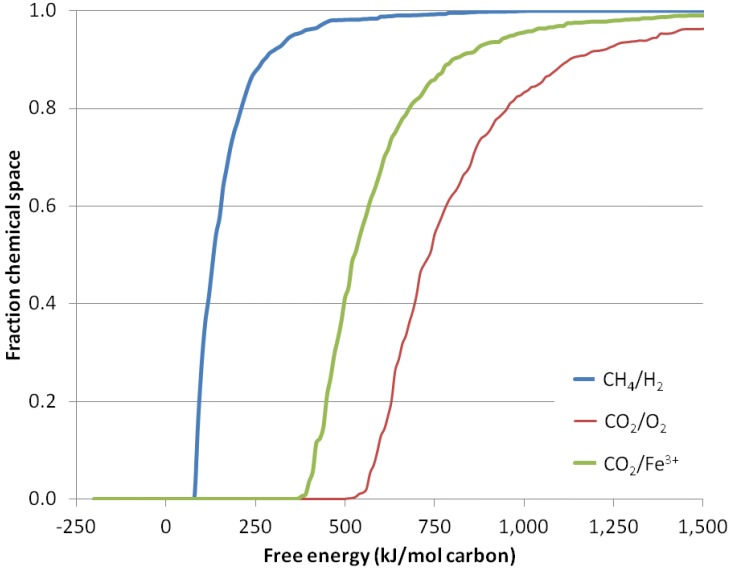
Chemical diversity accessible as a function of free energy. The fraction of the chemical space that can be captured for a given expenditure of energy. X-axis: input free energy. Y-axis: fraction of the 1,987,593 structures of nine or less non-H atoms generated by COMBIMOL that can be generated with no more than the free energy input on the X-axis. Shown are reactions where CH_4_ is oxidized, generating free H_2_ (blue), and CO_2_ is reduced in reactions generating free O_2_ (red) and Fe^3+^ (green).

We do not know, and for this exercise it is not relevant, what the fraction of the chemical space that life has to explore actually is. We only assume that the fraction of the chemical space is similar for life in any environment and that life will evolve from the compounds that require the least energy to make in its original environment [67]. Figure 2 presents the results of our analysis. In short, synthesizing a fraction of the chemical space from CO_2_ and H_2_O takes between five- and 10-times as much free energy as synthesizing the same fraction from CH_4_ and H_2_O. This is not materially affected by changing the concentrations of CH_4_ (between 10^−3^ bar and 10 bar), CO_2_ (between 10^−3^ bar and 10 bar) or the temperature (between 2 °C and 115 °C) (not shown).

The lower energy requirement for hydrogenic photosynthesis does not necessarily mean that a hydrogenic photosynthesis-based biosphere will have more biomass than an oxygenic photosynthesis-based biosphere. For surface photosynthesis on Earth, the limit on biomass productivity is usually access to CO_2_ or to other nutrients (especially water for land plants) (reviewed in [68]). Light energy is not a limiting factor. However, the lower energy requirement for hydrogenic photosynthesis may mean that photosynthetic life can be supported by environments with less light in them than on Earth. We discuss this concept in Section 3.6 below.

### 3.4. Electron Acceptors Other Than Hydrogen

We now address whether other electron acceptors than hydrogen could be the basis for photosynthesis on a CH_4_/H_2_-dominated planet. On Earth, anoxygenic photosynthesis uses environmental chemicals other than water as electron donors. Figure 2 illustrates the energy needed to capture CO_2_ using one such anoxygenic photosynthetic reaction, the oxidation of Fe^2+^ to Fe^3+^, a form of photosynthesis believed to be important to the early Earth. The energy required to drive this non-oxygenic photosynthesis is the energetics of oxygenic photosynthesis minus the energy released by the oxidation of iron by oxygen, so that the energy requirement of Reaction (7c) is the sum of the energy requirement of Reaction (7a) and the energy released by Reaction (7b).

Oxygenic reaction:3 CO_2_ + 4 H_2_O→C_3_H_8_ + 5O_2_ (7a)

Iron oxidation reaction:  2Fe^2+^ + ½O_2_ + 2H^+^→2Fe^3+^ + H_2_O (7b)

Overall:  3CO_2_ + 20Fe^2+^ + 20H^+^→C_3_H_8_ + 20Fe^3+^ + 6H_2_O (7c)

We can estimate the feasibility of analogous photosynthetic reactions in a CH_4_/H_2_-rich environment from the calculation of the energy available from the reduction of species that could be present in the crust as oxidized species (which could act as alternative electron acceptors to hydrogen) and which have a more positive electrode potential than hydrogen, are extremely abundant or both. It is possible that the crust of a planet with a highly-reduced atmosphere and mantle will only contain the elements listed in Table 2 as their most reduced species. If the elements in Table 2 are only present as reduced species, then their reduction will not be a potential source of energy for biomass building.

**Table 2:** Potential alternative electron sink reactions. Reduction of elements likely to be present in exoplanet crusts by molecular hydrogen. Left column, element. Second column, reaction with hydrogen. All compounds are in dilute aqueous solution in water, pH = 7, T = 25 °C; unless stated otherwise, hydrogen is at one atmosphere pressure and water is liquid. Third column, ΔG^0^ of that reaction. Right column, the reference for the thermodynamic data used.

Element	Reaction	Free Energy Change (kJ/mol)	Ref for Free Energy Data
Nitrogen	½ N_2_ + 1½ H_2_ → NH_3_	−62.61	[65]
	½ N_2_ + ½ H_2_ + H_2_O → NH_2_OH	+183.8	[69]
Phosphorus	H_2_ + HPO_4_^2−^ → HPO_3_^2−^ + H_2_O	+27.2	[70]
	H_2_ + HPO_3_^2−^ + H^+^ → H_2_PO_2_^−^ + H_2_O	+84.3	[70,71]
	½H_2_ + H_2_PO_2_^−^ + H^+^ → P_(s)_ + 2H_2_O	+52.8	[70,71]
	P_(s)_ + 1½H_2_ → PH_3_	+5.4	[70,72]
	Overall 4 H_2_ + HPO_4_^2−^ + 2H^+^ → PH_3_ + 4H_2_O	+169.8	
Sulfur	SO_4_^2−^ + H_2_ → SO_3_^2−^ + H_2_O	+12.45	[65]
	SO_3_^2−^ + 2H_2_ +2H^+^ → S_(s)_ + 3H_2_O	−248.29	[65]
	S_(s)_ + H_2_ → H_2_S	−44.81	[65]
	Overall SO_4_^2−^ + 2H^+^ + 4H_2_ → H_2_S + 4H_2_O	−280.8	
Iron	½ H_2_ + Fe^3+^ + OH^−^ → Fe^2+^ + H_2_O	−125.8	[65]
	H_2_ + Fe^2+^ + 2OH^−^ → Fe_(s)_ + 2H_2_O	−6.1	[65]
Manganese	Mn^3+^ + ½ H_2_ + OH^−^ > Mn^2+^ + H_2_O	−273.3	[65]
	H_2_ + Mn^2+^ + 2OH^−^ → Mn_(s)_ + 2H_2_O	−24.9	[65]
Silicon	2H_2_ + H_4_SiO_4 (s)_ → Si_(s)_ + 4H_2_O	+384.5	[71]
	2H_2(g)_ + SiO_2(s)_ > Si_(s)_ + 2H_2_O	+382.1	[71]
	Si_(s)_ + 2H_2(g)_ → SiH_4__(g)_	+56.9	[71]
Aluminium	3H_2 (g)_ + Al_2_O_3 (s)_ → 2Al_(s)_ +3 H_2_O	+871.0	[71]
Copper	Cu^2+^ + ½H_2_ → Cu^+^ + H^+^	−19.4	[65]
	Cu^+^ + ½ H_2_ → Cu_(s)_ + H^+^	−57.8	[65]
Vanadium	H_2_VO_4_^−^ +2H^+^ + ½H_2_ → HVO_2_^+^ + 2H_2_O	−113.8	[65]
	HVO_2_^+^ + ½H_2_ → VO^+^ + H_2_O	−243.5	[65]
	VO^+^ + ½ H_2_ → VOH^+^	17.5	[65]
	VOH^+^ + H_2_ → V_(s)_ + H^+^ + H_2_O	122.7	[65]

We review the potential anhydrogenic photosynthetic pathways that could be available to life. As an approximation, we can assume that the elements in the crust of any rocky planet will be similar to those in the crust and upper mantle of the Earth, as listed in [73,74,75]. Of these, many are more electropositive than hydrogen and, so, are implausible electron acceptors. We illustrate why with the following example. We might consider the reduction of copper to be a plausible anhydrogenic photosynthetic reaction, with the following overall chemistry,

CH_4_ + H_2_O + 4Cu^+^ → CH_2_O + 4Cu + 4H^+^ (8)

We could also imagine life reducing sodium ions to sodium metal, in an analogous reaction:

CH_4_ + H_2_O + 4Na^+^ → CH_2_O + 4Na + 4H^+^ (9)

However, while Reaction (8) may be a plausible alternative to Reaction (5), Reaction (9) is absurd. Reaction (9) would take far more energy than Reaction (5), and the sodium metal produced would immediately react with water, releasing that energy and hydrogen gas, as illustrated by the well-known reaction of sodium with water, which produces Na^+^ and H_2_ gas with so much energy that it melts the sodium and boils the water (see [76] for a large-scale demonstration of this chemistry). Elements substantially more electropositive than hydrogen are therefore not plausible electron acceptors. Elements substantially more electronegative than hydrogen, such as halogens, are already fully reduced in the planetary crust, and so cannot accept electrons to be reduced further: they are therefore also implausible electron acceptors.

Thus, the only plausible alternative sinks for electrons in a hydrogen-rich, methane-containing environment are Si and Al (because they are highly abundant) and Fe, P, Mn, S, N, V and Cu (because their reduction may be energetically more favorable than the production of hydrogen gas). Table 2 lists the chemistry of the reduction of these elements and the standard Gibbs free energy of their reduction by hydrogen. Many others elements can be reduced by hydrogen, but are rare, and so those elements are not likely to support a substantial biosphere (examples might include arsenic, silver, mercury). Aluminium and silicon are included in Table 2 for completeness, as Al and Si are the most abundant reducible species in the crust: however, the energies listed in Table 2 illustrate that they require large amounts of energy to reduce, and so they are extremely unlikely to be a substrate for reduced world photosynthesis.

Of the elements listed in Table 2, only sulfur and nitrogen can be reduced to volatile products, producing hydrogen sulfide and ammonia, respectively. Hydrogen sulfide is a poor biosignature gas, as it is expected to be generated by geochemical sources [51]. On a reduced planet, it is likely that hydrogen sulfide will be the dominant sulfur gas produced by outgassing. We have speculated before that ammonia could be a biosignature gas in an H_2_-dominated atmosphere: this analysis confirms that it is thermodynamically favorable to make ammonia. Phosphine has been claimed to be produced by terrestrial organisms in highly reducing environments [77,78]. We find that phosphorus compounds are not thermodynamically plausible photosynthetic side-products in the CH_4_/H_2_-dominated environment. The very high free energy of the reduction of phosphate also suggests that phosphine is an unlikely component of volcanic gases, no matter how reduced the mantle is.

We conclude therefore that hydrogen sulfide, ammonia or hydrogen are possible volatile photosynthetic waste products from photosynthesis in a hydrogen-dominated environment. Of these, only ammonia will be detectable remotely as a biosignature gas. Reduced metal salts or elemental metals may also be produced, but will be solids and, so, not detectable.

### 3.5. Photon Energies for H_2_-Dominated Photosynthesis

The previous sections addressed the relative amount of energy needed to drive biomass formation from methane in a hydrogen-rich environment and the thermodynamically plausible by-products of that photosynthesis. However, the energy must be provided by photons of the right wavelength. Photon energy is distinct from the total energy required to build biomass. On Earth, it takes 48 red photons to build one glucose molecule, but that does not mean that 24 blue photons or 96 NIR photons can build a glucose molecule, because each photon must have enough energy to drive the transfer of one electron to water. To adapt Hoehler’s analogy [62], total free energy is the current needed to drive the machinery of photosynthesis, and photon energy is the voltage. Both current capacity and voltage need to be sufficient to make the machinery work. In this section, we examine whether hydrogenic photosynthesis could be driven by photons that are likely to be available to life on the surface of a rocky planet.

As a starting point, we review the energetics of oxygenic photosynthesis. Oxygenic photosynthesis (Reaction (2)) results in the oxidation of water and the reduction of carbon dioxide. These two processes can be separated into two half-reactions:

½ H_2_O → ¼ O_2_ + H^+^ + e^−^ (10a)

¼ CO_2_ + e^−^ + H^+^ → ¼ CH_2_O + ¼ H_2_O (10b)

The reaction is written as two separate reactions in this way to make clear that this is a redox reaction (one that transfers electrons between molecules) and that the key energy-requiring step of the reaction is that splitting water to molecular oxygen and an electron. Anoxygenic photosynthesis using Fe(II) as an electron donor (Reaction (6)) can similarly be written:

Fe(OH)_2_ + H_2_O→Fe(OH)_3_ + H^+^ + e^−^ (11a)

¼ CO_2_ + e^−^ + H^+^ → ¼ CH_2_O + ¼ H_2_O (11b)

It is clear from this formulation that Reactions (10b) and (11b) are the same. The difference between Reaction (2) and Reaction (6) is what compound provides the electron, water (in Reaction (2)/(10a)) or ferrous iron (Reaction (6)/(11a)).

Half-reactions, such as Reaction (10a), have a characteristic energy, represented as a voltage (the standard electrode potential, E^0^) relative to the reduction of H^+^ to H_2_. In order to power oxygenic photosynthesis, life has to both collect enough energy in total per mole of carbon to at least compensate for the energy required to drive Reaction (2) and collect individual photons with at least enough energy to drive Reaction (10a).

For background, we will review how the energy needed to drive Reaction (10a) is calculated, before applying the same arguments to hydrogenic photosynthesis. The energy of a mole of photons is given by:

(B) $$D=\frac{hcA}{\text{λ}}$$

where D = energy in kJ/mol, h = Plank’s constant, c = speed of light, A = Avogadro’s constant and λ = the wavelength of the light. The energy of a reaction can be related to the voltage in an electrochemical cell made from a half-cell in which that reaction happens coupled to a standard H^+^/H_2_ cell by:

ΔG = −nFE^0^ (C)

where ΔG is the free energy of the reaction in kJ/mol, n is the number of electrons transferred, F is Faraday’s constant and E^0^ is the standard electrode potential of the half-cell. Thus, if a photon is used to drive a reaction generating a free electron (such as Reaction (10a) or (11a)) and that electron is used to power a thermodynamically unfavorable reduction, then the maximum wavelength of light (*i.e.*, photons of the minimum energy) that can power that reaction is given by:

(D) $$\lambda =\frac{hcA}{F\cdot E^{0}}$$

The reason that Equation (D) estimates a maximum wavelength is as follows. Equation (C) relates the free energy released by a small change of the reaction while it is at equilibrium. Thus, Equation (C) calculates the energy needed to bring Reaction (2) to equilibrium in a completely efficient system. However, we wish to drive the reaction to completion, not to equilibrium, and no real-world macroscopic system is 100% efficient. To achieve complete reaction in an inefficient system, more energy is needed, represented by an additional voltage, called an overvoltage. In oxidative phosphorylation (another complex series of reactions capturing environmental energy), the voltage required to synthesize ATP from the translocation of four protons (0.15 V) is driven by a typical mitochondrial membrane potential of 0.2 V, an overvoltage of ~30% [79,80]. For oxygenic photosynthesis, Reaction (11a) has an energy corresponding to 1.23 V (~1000 nm photons). Chloroplasts cannot generate oxygen using light longer than 680 nm (1.8 V) (known as the “red drop” effect, reviewed in [81]), but some cyanobacteria can use light of 720 nm (1.68 V) in oxygenic photosynthesis, again an overvoltage of ~30% [82]. By contrast, longer wavelengths can be used to oxidize sulfur or Fe^2+^ in anoxygenic photosynthesis [64]. The overvoltage values are similar to the overvoltage needed to power the electrolysis of water for commercial hydrogen generation (typically +30% of the thermodynamically minimum voltage; reviewed in [83]).

We now apply this background understanding to the case of hydrogenic photosynthesis. We emphasize that this is speculative, but the speculation is useful in comparing hydrogenic photosynthesis on our hydrogen-dominated exoplanet to oxygenic photosynthesis on Earth.

The half-cell reactions for hydrogenic photosynthesis (Reaction (5)) are:

CH_4_ + H_2_O→CH_2_O + 4H^+^ + 4e^−^  E^0^ = 0.63 V (12a)

4H^+^ + 4e^−^→2H_2  _E^0^ = 0 V (12b)

The voltages will be affected by pH, but because the overall reaction (Reaction (5)) does not produce or consume H^+^, the effects of pH on Reactions (12a) and (12b) will cancel.

**Figure 3:**
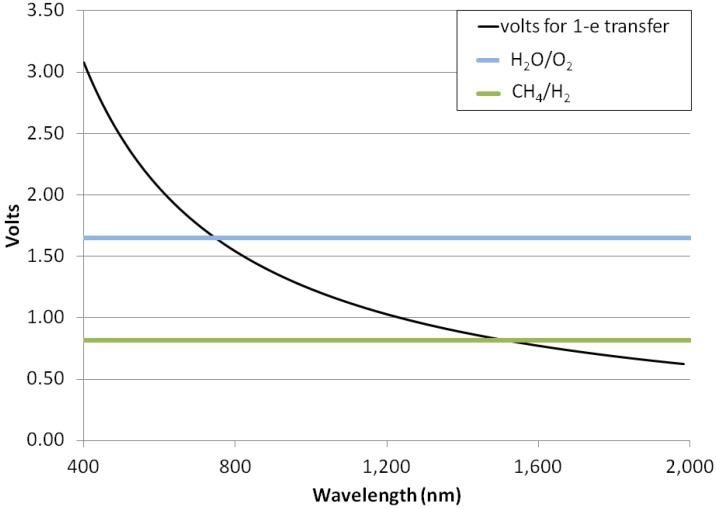
Photon energies for photosynthesis. Illustration of the maximum wavelengths that might be required for different types of photosynthetic reactions. Y-axis, volts. X-axis, the wavelength of light (nm). The black curve shows the standard electrode potential of a single electron reaction that consumes (or in the reverse direction, generates) energy equivalent to the energy in a mole of photons of a particular wavelength. Horizontal bars show the standard electrode potential needed to drive the generation of free oxygen from water and free hydrogen from CH_4_ + H_2_O. The point where each horizontal bar crosses the black curve illustrates the likely maximum wavelength that could be used to power the relevant reaction.

If hydrogenic photosynthesis with the overall stoichiometry of Reaction (5) follows a similar pattern to terrestrial photosynthesis and requires a ~30% overvoltage, then we can expect that it will be driven by photons capable of driving a half-cell with a standard electrode potential of ~0.82 V, which are photons with a wavelength of no more than ~1500 nm (Figure 3). Shorter wavelength (more energetic) photons can also be used, with the excess photon energy dissipated as heat. This is a substantially longer wavelength light than can be used for oxygenic photosynthesis and longer than is observed for any photosynthesis on Earth. If no overvoltage is required or if two or more photon absorption events are coupled to one oxidation, then photons of even longer wavelengths could be utilized in hydrogenic photosynthesis. We note that, if photosynthesis using wavelengths of 1000–1500 nm does occur on worlds with hydrogen-rich atmospheres, then these worlds’ biospheres will not exhibit the “red edge” that is so striking a spectral feature of terrestrial vegetation [39], as photosynthetic organisms on those worlds will preferentially absorb NIR radiation to carry out biomass building.

We note that this discussion solely addresses the photon energies needed to drive photosynthesis. Other chemistry might limit the photons used by life in a hydrogen-rich environment, such as atmospheric absorption of a chemically ideal wavelength or damage to the photosystem by absorption of high energy photons [41].

### 3.6. Planetary Environments for Hydrogenic Photosynthesis

We can now explore what planetary environments might provide the necessary flux of photons of the necessary wavelengths to sustain photosynthesis. We present order of magnitude estimates to illustrate that photosynthesis in a hydrogen-dominated atmosphere is feasible. First, we search the literature to determine the minimum light flux for Earth photosynthesis. Then, we construct a grid of stellar and planetary parameters and build hydrogen-dominated vertical atmosphere profiles with surface temperatures of 25 °C. We calculate the photosynthetically-active energy flux that reaches the surface (assuming a cloud-free atmosphere). Finally, we determine what planetary and stellar properties have the strongest impact on the photon flux and what environment is expected to be suitable for hydrogenic photosynthesis. We show that hydrogenic photosynthesis at a surface temperature of 25 °C is plausible, if the surface pressure is less than 30 bars. Such planets orbit at a maximum of ~0.5 AU to ~10 AU around M dwarfs or Solar-like stars, respectively. We note that these distances are well outside the conventional “habitable zone”, because of the very strong greenhouse effect of CIA in H_2_-dominated atmospheres.

The minimum photon flux for Earth photosynthesis varies with the life forms and environments studied. Photosynthesis has been seen in organisms growing in the red and NIR light given off by black smoker vents on the ocean floor, where the light flux is observed to be 6 × 10^13^ photons/cm^2^/s [84]. Laboratory studies and *in situ* measurements show that photon fluxes as low as 10^12^–5 × 10^10^ photons/cm^2^/s can sustain photosynthesis in bacteria isolated from the Black Sea at the chemocline in December [85]. We therefore chose 10^13^ photons/cm^2^/s as a threshold for the flux of photons necessary to sustain photosynthetic life. This is 1–2 orders of magnitude above the minimum flux found to be used by photosynthesizing organisms on Earth, and it is roughly two orders of magnitude below the average photon flux impacting the surface of Earth. This is therefore a conservative estimate, especially as photosynthesis in a reducing environment is likely to need five- to 10-times less total energy than in an oxidizing environment.

We use the all-troposphere approximation to simulate the atmospheric temperature-pressure profile, and we perform inverse climate modelling (see [47,86] for more details). Only the troposphere is studied, because the stratosphere and other atmospheric regions are mostly optically thin in the optical and near-infrared. Thus, their impact on the surface fluxes are negligible. Inverse climate modelling means that we construct a convective pressure-temperature profile with a surface temperature of 25 °C (other planetary parameters are varied as discussed below), and we search for the semi-major axis where the atmosphere is in radiative equilibrium. The set surface temperature allows us to narrow down the parameter space and to focus on planets with favorable surface climates for life.

We use a simplified model troposphere consisting solely of H_2_ and H_2_O. For radiative balance calculations, H_2_ is the relevant species. In an H_2_-dominated atmosphere, the average molecular weight (which determines the scale height) will be determined by H_2_. The greenhouse effect of the atmosphere is dominated by H_2_-H_2_ CIA, and constituents that have a significant greenhouse effect on Earth, such as CH_4_ and H_2_O, make only a minor contribution to the greenhouse effect in an H_2_-dominated atmosphere [47]. Water vapor is included for consistency with the existence of surface water, but makes a minor contribution to the radiative balance.

A wide range of stellar and planetary properties are considered, and we find that the stellar mass and the surface pressure have the strongest impact on the surface photon flux. The stellar mass is varied between 0.1 and 1 solar mass (equivalent to an effective temperature range of 3000–6000 K), and the surface pressure of the planet’s atmosphere ranges between 0.1 and 300 bars. We performed simulations where other parameters were varied (such as relative humidity, surface gravity and surface albedo), but we found that their effect on the surface photon flux is small. Thus, in the following, the relative humidity is set to 50% (corresponding to a surface mixing ratio of 0.01), the surface gravity is 20 m/s^2^ (roughly twice Earth’s) and the surface albedo is 0.2 for simplicity.

**Figure 4:**
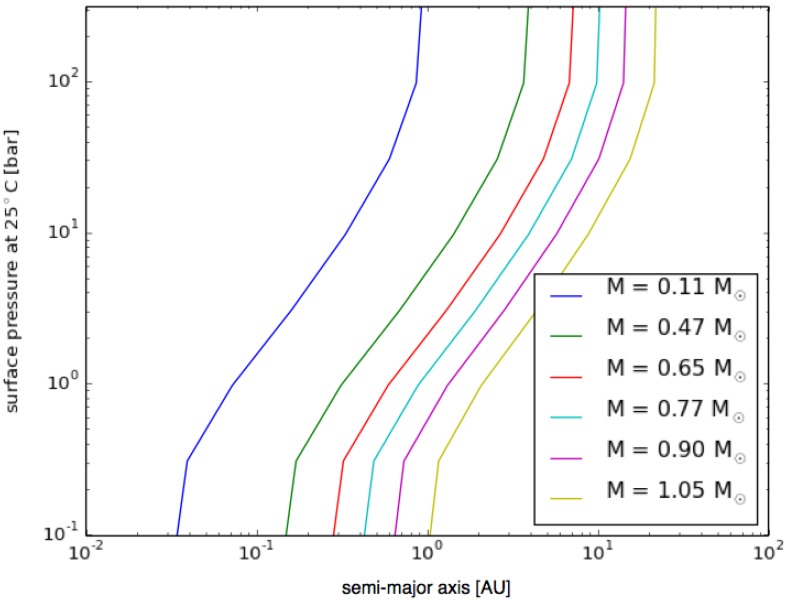
Pressure *vs.* orbital parameters for planets around different stars. Plot of the surface pressure (Y-axis) needed to maintain a surface temperature of 25 °C on a planet with a 20-m/s^2^ surface gravity, 90% H_2_ atmosphere, orbiting around different mass stars in a roughly circular orbit with a specific semi-major axis (X-axis). See the text for other conditions. The different color lines represent different stellar masses: higher masses of atmosphere (*i.e.*, higher surface pressure) mean that a planet must orbit further from its star to have a surface temperature of 25 °C, and a higher mass star also means that a wider orbit is required.

Figure 4 shows the semi-major axis and surface pressure where the atmosphere is in radiative equilibrium with a surface temperature of 25 °C around various main sequence stars. The figure illustrates that hydrogen is an effective greenhouse gas due to collision-induced absorption (see, e.g., [48]). If the surface pressure is low, the greenhouse effect is negligible and only close-in planets can maintain a surface temperature of 25 °C. If the atmosphere is more substantial, the surface temperature remains favorable for life even beyond 10 AU around a Solar-like star.

**Figure 5:**
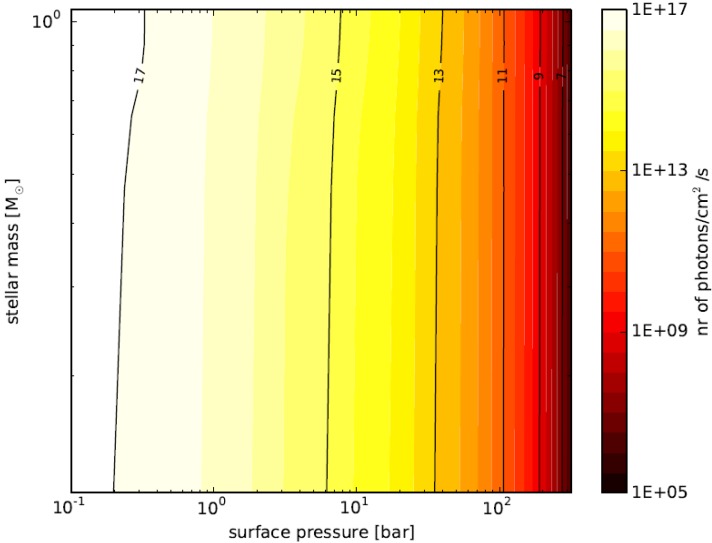
Surface photon flux as a function of surface pressure and stellar mass. For a combination of stellar mass (Y-axis) and surface pressure (X-axis), the semi-major axis of the planet was calculated as per Figure 4. From the stellar photon flux, distance and atmospheric absorption, the surface flux of photons was calculated (color scale on the right of the graph). Stellar mass has a minimal effect, because a higher stellar mass requires the planet to orbit further from the star to maintain a clement surface environment.

We calculate the flux that reaches the surface short-ward of 1.5 microns in units of photons/cm^2^/s, and the result is illustrated in Figure 5. The X-axis represents the surface pressure, the Y-axis is the stellar mass, and the contour levels show the surface flux. There is no need to show the semi-major axes, because it can be uniquely determined from Figure 4. We see in Figure 5 that the photon flux is larger than our critical value (10^13^ photons/cm^2^/s), if the surface pressure is less than 30 bars. The contour lines are almost vertical, but it is slightly inclined to the right for high-mass stars. That is because solar-like stars produce more photosynthetically-active photons than M dwarfs.

We conclude that hydrogen photosynthesis is plausible from a radiative transfer point of view. Sufficient photons arrive to the surface to drive photosynthesis, even though habitable planets with hydrogen-dominated atmospheres are generally located at larger distances from the host star than an Earth-like planet with an N_2_/O_2_ atmosphere. As the relative numbers of planetary masses, orbits and atmospheres is not (yet) known, we cannot estimate the frequency of planets on which hydrogenic photosynthesis is plausible, but the calculations summarized in Figure 4 and Figure 5 suggest that they are no less likely than Earth-like planets with oxidized atmospheres and clement surface temperatures, and it is not implausible that they are more common [49].

## 4. Discussions

Photosynthesis provides nearly all of the energy for the synthesis of biomass on Earth. The evolution of the ability to capture electromagnetic energy was a pivotal step in the development of life on Earth and has occurred at least three times independently. The ability to use water as a source of electrons for photosynthesis was also key to creating the abundant biosphere we walk in today. The evolution of oxygenesis seems to have happened only once. The products of oxygenic photosynthesis—O_2_ gas and its photolytic product, ozone—are considered the most distinctive chemical biosignatures of life on Earth [38].

On a planet with a reduced surface and a reduced atmosphere, if carbon is present overwhelmingly as methane, then the chemistry of photosynthesis will necessarily be different from the chemistry of terrestrial photosynthesis. Methane will have to be oxidized to build biomass, and an environmental chemical will consequently have to be reduced. The energy captured from photons will drive this process. If the atmosphere of a planet is reduced and the surface is oxidized, then life can also capture energy from the redox gradient between the atmosphere and the surface minerals. On Earth, such disequilibria are rare and are rapidly exploited by life. If the atmosphere contains both oxidized and reduced species (CO_2_ and H_2_), again, life will rapidly exploit this thermodynamic disequilibrium to remove the CO_2_ and replace it with CH_4_.

In this paper, we have provided some suggestions concerning the possible chemistry, energetics and photochemistry of photosynthesis that generates hydrogen (which we have termed “hydrogenic” photosynthesis). Conclusions from our analysis are that:

(1) Hydrogenic photosynthesis requires 5 to 10 times less energy to build a given mass of biomass from methane as oxygenic photosynthesis requires to build the same amount of biomass from carbon dioxide;
(2) Hydrogenic photosynthesis could be driven by photons into the near-infrared—1500 nm—whereas oxygenic photosynthesis is observed to be powered only by red photons of a wavelength ≤720 nm.
(3) Planets with surface conditions suitable for hydrogenic photosynthesis may exist over a much wider range of orbital parameters than the conventional habitable zone;
(4) Hydrogen gas is the most plausible reduced product of methane-oxidizing photosynthesis. Ammonia gas may also be a photosynthetic waste product. Other waste products can be suggested, but either require more energy to make or require rare starting materials and, in any case are very unlikely to be detectable remotely.

This has implications for the probability and detectability of photosynthesizing life on super-Earths with hydrogen-dominated atmospheres.

### 4.1. Limited Biosignature Gases from Hydrogenic Photosynthesis

The principal biosignature gas of life on Earth is oxygen, the product of photosynthesis. High atmospheric oxygen is a highly distinctive sign of life (see [38] and the references therein). The chemically equivalent by-product gas from hydrogenic photosynthesis is hydrogen gas. However, this will not be detectable, as (by definition) our exoplanet is modelled as a planet with abundant hydrogen in its atmosphere.

This does not mean that life itself would be undetectable on a rocky planet with a hydrogen-rich atmosphere. We have previously modelled the detectability of a range of potential biosignature gases in the hydrogen-rich atmospheres of rocky planets [52]. Biosignature gases can be classified as Type I (made by reactions that capture energy from environmental energy gradient), Type II (gases made as side-products of biomass construction, such as the reactions considered in this paper) and Type III (gases made from other aspects of metabolism) (see [68] for more details of this classification). Several Type I biosignature gases that are quite characteristic of life on Earth are not useful biosignatures on a planet with a hydrogen-dominated atmosphere, either because they are naturally made by geochemical processes or because they are rapidly destroyed by photochemical processes. Only ammonia has been suggested to be a useful Type I biosignature on an H_2_-dominated exoplanet [52], although even this is not completely diagnostic of life, as it is possible for trace amounts of ammonia to be made by hydrothermal processes (for terrestrial examples of this, see [87,88,89]). While ammonia is photolyzed readily, only small fluxes are required to maintain a detectable level of ammonia in a hydrogen-rich atmosphere [52]. Here, we show the same, negative conclusion is true of photosynthetic Type II biosignatures. Of the gases that might be made, hydrogen is already present in the atmosphere, and hydrogen sulfide will not be distinct from volcanic gases (H_2_S, or the product of volcanic SO_2_ reacting with atmospheric H_2_). Other possible photosynthetic products are solids and would not be detectable remotely.

We conclude that the only biosignature gas that is likely to be detectable from chemosynthesis or photosynthesis in a hydrogen-dominated atmosphere is ammonia. While highly suggestive of the presence of life, ammonia is not robust evidence for life. For such a planet, the absence of atmospheric evidence for life is not evidence for the absence of life.

### 4.2. Evolution of Photosynthesis in an H_2_-Dominated Environment

Terrestrial metabolism gives us optimism that life can evolve hydrogenic photosynthesis at least as easily as anoxygenic photosynthesis was evolved on Earth. This is because both of the components of hydrogenic photosynthesis have evolved on Earth:

(1) Capture of light energy and its use to generate H_2_ gas;
(2) Oxidation of methane to generate H_2_.

A wide range of organisms, including purple bacteria and eukaryotic algae, can generate hydrogen from photosynthesis (reviewed in [90,91,92]). However, these organisms fix CO_2_, not methane; H_2_ is generated either as part of non-growing energy metabolism or for nitrogen reduction [93]. Therefore, photosynthetic hydrogen generation by terrestrial organisms is not an example of Reaction (5). Hydrogenesis is believed to have evolved very early in life’s evolution: hydrogenase, the key enzyme in H_2_ production, probably evolved before photosynthesis [94].

In anoxic sediments where the concentration of H_2_ is kept low, organisms are believed to carry out the reaction:

CH_4_ + 2H_2_O→CO_2_ + 4H_2_ (13)

as a reversal of the more usual methanogenic reaction (Reaction (3) above) to capture chemical energy [95]. Reaction (13) would usually be energy-consuming: it only yields energy in these sediments because other organisms remove the H_2_ by oxidizing it with sulfate [95,96,97], thus:

4H_2_ + SO_4_^2−^→HS^−^ + 3H_2_O + OH^−^ (14)

so the overall process is [96,98]:

CH_4_ + SO_4_^2−^→HCO_3_^−^ + HS^−^ + H_2_O (15)

Thus, on Earth, energy-consuming Reaction (13) is coupled to energy-yielding Reaction (14) to drive overall Reaction (15). Reaction (14), the reduction of sulfate, is probably also a very ancient metabolic pathway on Earth [99]. Thermodynamically, there is no reason that the organisms could not couple photosynthetic energy capture to drive Reaction (13) or its biomass capture equivalent, Reaction (4). The reason that this does not occur on Earth might be that on Earth, Reactions (13)–(15) only occur in black anoxic sediments where there is no light. It would be interesting to consider whether there are any hydrogen-rich, light-accessible environments on Earth where photosynthetic production of hydrogen and Reaction (13) could occur together.

From a purely human point of view, the evolution of hydrogenic photosynthesis might be a disappointing discovery on another world, for reasons implicit in Figure 1. Just as making biomass in an oxidized environment requires more energy, breaking down biomass in an oxidized environment releases more energy. In particular, oxidizing biomass using molecular oxygen releases substantially more energy than reducing it using molecular hydrogen. A commonly-held explanation for the rise of complex animals in the late Pre-Cambrian and Cambrian periods was the rise in atmospheric oxygen that allowed their energy-intensive lifestyles [15].

Does a hydrogen-rich atmosphere, reduced surface and concomitant hydrogenic photosynthesis therefore preclude complex animal life? We believe that such a pessimistic conclusion would be premature for two reasons.

Firstly, the association of the Cambrian explosion with the rise in atmospheric oxygen is only an association. Many animals can live for extensive parts of their life cycle without oxygen (reviewed in [100,101]), and recently, a group of obligate anaerobic invertebrates was discovered that lived entirely without O_2_ (albeit small and relatively inactive ones) [102]. These examples show that animal life may be possible at much lower energy levels than our own.

Secondly, oxidative metabolism yields so much energy because of the food it has to oxidize. Carbohydrates and fats are the storage materials that plants and animals chose to use exactly because they are the most efficient ways of storing energy in an oxidizing environment. However, they are not the only storage option. Predatory plankton preying on phytoplankton gain substantial energy from the metabolism of dimethylsulfonium proprionate (DMSP), releasing dimethyl sulfide (DMS) in large amounts [103,104]. DMSP is accumulated for reasons other than energetics (no one has convincingly argued what those reasons are [104,105,106]). Its energy of hydrolysis would be the same in oxidizing or reducing environments. In a reducing environment, highly oxidized compounds could be stored as energy storage materials, having the highest energy density when reduced with hydrogen, or other compounds with roles comparable to DMSP could be accumulated and be used as high-energy food. The absence of oxygen does not therefore preclude the possibility that other biomass components could be metabolized to yield lots of energy per gram.

## 5. Summary and Conclusions

We have described the likely features of photosynthesis on a rocky planet with a thin, hydrogen-dominated atmosphere. We argue that, in the presence of life, such a planet is likely to accumulate methane in its atmosphere and that biomass building in this environment necessarily requires the generation of a reduced side-product. A likely side-product is hydrogen gas. Other, less likely products include H_2_S, reduced metals or metal salts, and ammonia.

We have examined the energetics of possible photosynthetic processes and shown that they are plausible, and are likely to require less energy per unit biomass than photosynthesis on Earth.

We have shown that light of up to a 1500-nm wavelength can power this photosynthetic reaction. The lower energy requirements and longer wavelength light requirements mean that photosynthesis may be able to support a biosphere on planets much further from their star or with much denser atmospheres than is true for Earth-type, oxygenic photosynthesis. We have shown that a range of planets that can provide the right combination of surface pressure, temperature and photon flux are realistic around stars from 0.1–1 solar mass. However biospheres on those planets will be difficult to detect. Of the possible waste products of methane/hydrogen-based photosynthesis, only ammonia is a remotely detectable signature of biological activity, and even ammonia is not a robust signature of life, as geochemical processes could also produce ammonia. We speculate that hydrogenic photosynthesis could evolve at least as easily as anoxygenic photosynthesis has evolved on Earth and is not necessarily incompatible with the development of complex life.
